# Observation of nanoscale opto-mechanical molecular damping as the origin of spectroscopic contrast in photo induced force microscopy

**DOI:** 10.1038/s41467-020-19067-3

**Published:** 2020-11-10

**Authors:** Mohammad A. Almajhadi, Syed Mohammad Ashab Uddin, H. Kumar Wickramasinghe

**Affiliations:** 1 Department of Electrical Engineering and Computer Sciences, University of California, Irvine, CA 92697 USA; 2 Department of Electrical Engineering, University of Hail, Hail, 2440 Saudi Arabia

**Keywords:** Infrared spectroscopy, Optical physics, Microscopy, Atomic and molecular interactions with photons

## Abstract

Infrared photoinduced force microscopy (IR-PiFM) is a scanning probe spectroscopic technique that maps sample morphology and chemical properties on the nanometer (nm)-scale. Fabricated samples with nm periodicity such as self-assembly of block copolymer films can be chemically characterized by IR-PiFM with relative ease. Despite the success of IR-PiFM, the origin of spectroscopic contrast remains unclear, preventing the scientific community from conducting quantitative measurements. Here we experimentally investigate the contrast mechanism of IR-PiFM for recording vibrational resonances. We show that the measured spectroscopic information of a sample is directly related to the energy lost in the oscillating cantilever, which is a direct consequence of a molecule excited at its vibrational optical resonance—coined as opto-mechanical damping. The quality factor of the cantilever and the local sample polarizability can be mathematically correlated, enabling quantitative analysis. The basic theory for dissipative tip-sample interactions is introduced to model the observed opto-mechanical damping.

## Abstract (web summary)

Existing high-dimensional optical imaging techniques that record space and polarization cannot detect the photon’s time of arrival due to the limited speeds of electronic sensors. Here, the authors develop a single-shot ultrafast imaging modality to record light-speed high-dimensional events with picosecond resolution.

## Introduction

**Fig. 1:**
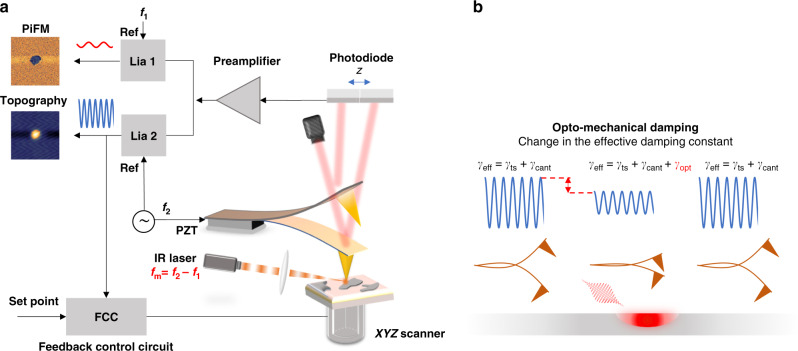
Schematic of IR PiFM experiment and principle of opto-mechanical damping. **a** The cantilever is mechanically vibrated at its second mechanical eigenmode *f*_2_, so that peak–peak oscillation is 6 nm. Lock-in amplifier and feedback laser position sensitive detector (PSD) are used to stabilize the cantilever nanometers from sample surface. The IR source is electrically triggered at *f*_m_ = *f*_2_ − *f*_1_, where *f*_1_ is the first mechanical eigenmode of the cantilever. The incident infrared pulse is p-polarized (along the tip axis) and focused to 20-μm-diameter spot. The topography and the PiFM signals are simultaneously recorded at *f*_2_ and *f*_1_, respectively. The image is generated via raster-scanning the sample under the tip. **b** illustrates the principle of opto-mechanical damping, where oscillation amplitude is damped due to IR vibrational resonance.

The integration of atomic force microscopy (AFM) with focused lasers has enabled nano-chemical imaging and spectroscopy with spatial resolution well beyond the diffraction limit. One classic example is apertureless near-field scanning optical microscopy^1–4^. In this method, the enhanced optical field of the scanned AFM probe is perturbed by the local near field generated by the excited sample, and the scattered near field (amplitude and phase) is detected in the far field using an interferometer to record the image. Photothermal-induced resonance^5,6^ and peak force infrared (IR)^7^ are two examples for characterizing sample chemical properties based on AFM. In these techniques, the sample thermal expansion induced by optical absorption is detected using an AFM tip in contact. An alternative, noninvasive microscopy and spectroscopy technique that has emerged recently is photoinduced force microscopy (PiFM)^8^ (Fig. 1). In this method, the tip–sample optical interaction is measured with the AFM operating in non-contact mode. The topography is recorded using the second mechanical eigenmode of the cantilever at *f*_2_. A quantum cascade laser (QCL) is amplitude modulated at *f*_m_ (where *f*_m_ = *f*_2_ − *f*_1_) and focused on the tip end, and the opto-mechanical response is measured at the first mechanical eigenmode at *f*_1_. Many applications of PiFM have emerged. Near-field electromagnetic field characterization^9–14^, nonlinear optical measurements such as Raman^15^ spectroscopy and stimulated Raman spectroscopy^16,17^, time-resolved pump-probe microscopy^18^, organic solar cell studies^19^, optical phonon polariton imaging, and nanoscale chemical imaging in the mid-IR^20^ are but a few examples.

While the dipole–dipole force model provides excellent agreement with the electromagnetic near-field measurements in the visible^14^ and with mid-IR plasmonic resonance spectra^21^, extending this model to IR vibrational resonances causes discrepancies between experiment and theory^20,22,23^. In particular, the dipole–dipole force model predicts a dispersive spectral response, while the experimental results show a purely dissipative response. Three alternative proposals for explaining PiFM spectroscopic contrast in the IR have been proposed to address this discrepancy. They are (1) detecting photothermal expansion using short-range repulsive forces acting on the AFM cantilever/tip^24,25^ in contact, (2) detecting photoacoustic pressure waves generated at the sample surface resulting in long-range repulsive forces acting on the cantilever/tip^25^, and (3) detecting van der Waals (vdW)-mediated force modulation caused by sample thermal expansion^26^.

In this paper, we report on a series of experiments aimed at unraveling the origin of PiFM spectroscopic contrast in the IR. Our experimental findings support the hypothesis that the spectroscopic contrast in PiFM is mediated by opto-mechanical damping of the cantilever oscillation as the optical wavelength is scanned through optical resonance. Here the rate of dissipated mechanical power due to interactions induced by tip–sample dissipation processes can be divided into three components: *γ*_ts_ due to adhesion, viscous damping, etc.; *γ*_cant_ due to air or fluid damping; and *γ*_opt_ due to opto-mechanical damping driven by dissipative near-field optical interaction. In our analysis, we assume that the opto-mechanical damping constant can be described by a velocity-dependent term in the Hamiltonian (as will be discussed later). The effective damping constant (*γ*_eff_) includes all damping effects (*γ*_eff_ = *γ*_ts_ + *γ*_cant_ + *γ*_opt_), i.e., the total mechanical power dissipation increases upon optical absorption. We hypothesize that opto-mechanical damping force (change in energy with respect to distance) is caused by the excited sample molecules creating a dissipative force on the vibrating tip. We show that this contrast mechanism provides an excellent match with the experimental results. The theory can be extended to the single monolayer detection limit.

## Results

### Distinguishing between repulsive and attractive optical forces

**Fig. 2:**
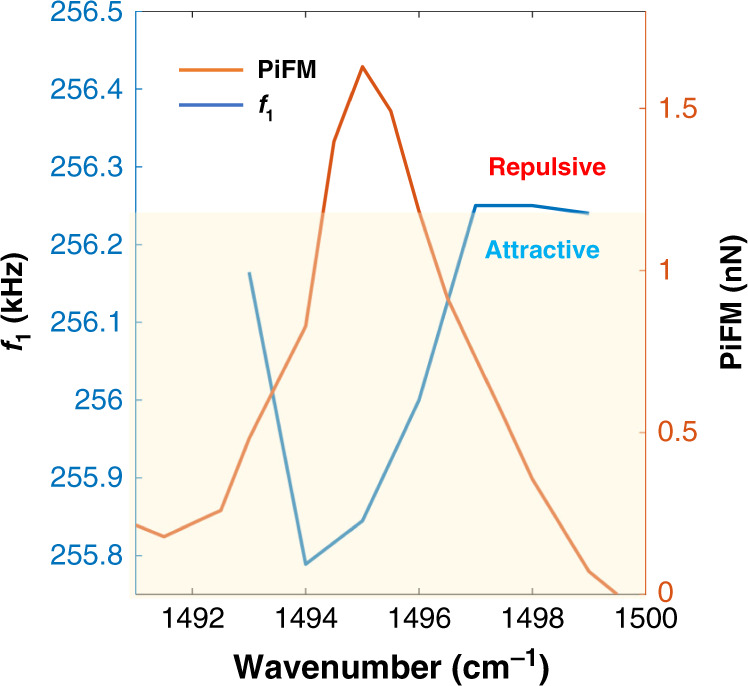
Cantilever frequency shift across an absorption band. Shift of the cantilever resonance frequency at *f*_1_ (blue line) across PS absorption band at 1495 cm^−1^ (orange line). The sample is 60-nm-thick PS film on Au substrate. Input average power was 1 mW focused to 20-μm-diameter spot.

Force gradients acting on an AFM tip shifts its dynamic stiffness (*k*) and resonance frequency *f*. Figure 2 shows results from a 60-nm-thick polystyrene (PS) film on gold (Au) substrate. We record the frequency shift of the cantilever at *f*_1_ while the tip–sample gap is controlled at *f*_2_. We plot the frequency shift at *f*_1_ as we scan the optical excitation wavelength through the PS resonance. When sample is excited on resonance, the frequency of the first eigenmode shows a maximum shift toward lower frequency values, relative to off resonance excitation, revealing the attractive nature of the optical force. Previous works^27^ have also come to the same conclusion, where the frequency shift was measured relative to the free oscillation amplitude (i.e., 3 μm away from the sample surface); however, in those experiments, non-optical effects, such as van der Waalʼs (vdW) forces, were not subtracted. In our experiments, we automatically eliminate any vdW force gradient effects by measuring the frequency shift at *f*_1_ as we scan through optical resonance while the tip is engaged. The observed frequency shifts were in the range of 500 Hz. Therefore, short-range thermal expansion forces in contact (repulsive forces) are not relevant in PiFM.

It is well known that energy absorbed at the surface of a sample can generate acoustic pressure waves in the surrounding gas—photoacoustics^28^. For our system, the pressure waves will have a wavelength ranging from 248 μm to 1.3 mm (corresponding to *f*_m_ = 1.38 MHz and *f*_1_ = 256 kHz). During one cycle of cantilever oscillation, the change in near-field photoacoustic force, i.e., acoustic force gradient, acting on the cantilever should be much smaller than the near-field optical force gradient acting on the tip. Photoacoustics generated by the 20 μm IR spot on the sample could still exert a global repulsive force on the cantilever. We were indeed able to detect a global photoacoustic effect originating from the focused IR beam for relatively thick samples (>200 nm) (see Supplementary Fig. 1) but only when the tip is retracted a few μm from sample when the much larger optical forces become negligible. We also observed that the global photoacoustic signal disappears when the system is operated in a vacuum of 0.3 torr. Based on these considerations, we conclude that the near-field repulsive force due to gas photoacoustics will have minimal effect on the overall near-field PiFM signal in our measurements. Our experimental observations have refuted the proposals that gas photoacoustic forces or short-range thermal expansion forces play any significant role in PiFM contrast, at least in the regime that we have investigated—i.e., organic samples with thicknesses <60 nm. We will therefore no longer consider photothermal expansion or gas photoacoustics as potential contributors to hetrodyne PiFM contrast mechanism.

### Piezo vibration experiments and PiFM sensitivity to thermal expansion

We showed that thermal expansion and photoacoustics (short- and long-range repulsive forces, respectively) do not play a significant role in our PiFM set-up. This leaves us with thermally modulated vdW forces ($F_{{\mathrm{th}}}^{{\mathrm{vdW}}}$), i.e., thermal expansion modulates and amplifies the vdW force, which in turn acts on the AFM tip and consequently generates the PiFM signal. The modulated $F_{{\mathrm{th}}}^{{\mathrm{vdW}}}$ are long-range attractive forces. In this section, we mimic thermal expansion in our set-up by vibrating a mirrored lead zirconium titanate (PZT) crystal—which in turn modulates the vdW force. Our PZT crystal was independently calibrated using a heterodyne laser interferometer (see Supplementary Fig. 2). Experiments were carried out to determine the smallest detectable thermal expansion in our PiFM.

**Fig. 3:**
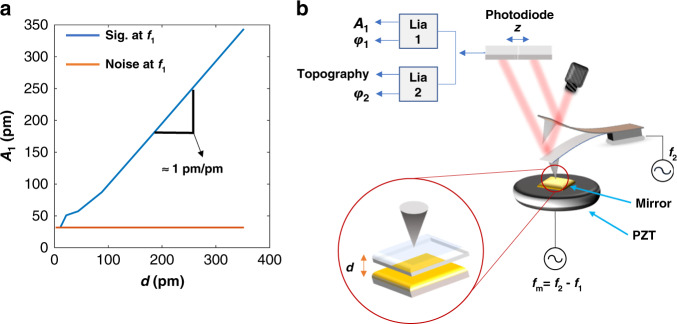
Non-contact AFM sensitivity to thermal expansion. **a** The peak–peak oscillation amplitude (blue solid line) measured at *f*_1_ as a function of the PZT displacement amplitude (*d*). The noise level of the system measured at *f*_1_ corresponds to oscillation of about 32 pm peak–peak as indicated by the orange line. Tip–sample gap is controlled at *f*_2_ and the PZT is driven at *f*_m_ = *f*_2_ − *f*_1_. **b** depicts the experimental set-up.

Figure 3b depicts our experimental set-up. Template-stripped gold (TSG) attached to PZT was vibrated at *f*_m_ = *f*_2_ − *f*_1_. The modulated vdW at *f*_m_ mixes with *f*_2_ to generate a signal at *f*_1_ due to nonlinear tip–sample interactions. We plotted the sensitivity (*S*) defined as the ratio of the measured signal (pm) to the piezo displacement (pm) and compared it with the noise level of the PiFM to determine the minimum detectable thermal expansion. Results in Fig. 3a show a linear relationship between PZT displacement and the observed signal, with *S* = *A*_1_/*d* = 1 pm pm^−1^, where *A*_1_ is the peak–peak oscillation amplitude of the first mechanical mode and *d* is the PZT displacement as shown in Fig. 3a (Fig. 3a will be used later to estimate the thermal expansion contribution to our PiFM signal in our monolayer experiments). Since the noise level measured at *f*_1_ is about 32 pm for 5 ms integration time (orange line in Fig. 3a, and see also Supplementary Fig. 4), thermal expansion below 32 pm will not be detectable in our system. Thermal expansion for a 60-nm PS film on silicon substrate (excited at 1452 cm^−1^ with 5 mW average power focused to 20-μm-diameter spot) has been previously calculated to be about 30 pm, which is already below our noise level^26^. That study shows that thermal signals generated by monolayer samples with typical thermal expansion of a few pm would be barely detectable. In the following section, the response of a 4-methylbenzenethiol (4-MBT) monolayer on TSG was measured with a signal-to-noise (S/N) ratio of 100, further confirming that our PiFM contrast cannot be thermal or $F_{{\mathrm{th}}}^{{\mathrm{vdW}}}$ in origin.

### Monolayer PiFM experiments

**Fig. 4:**
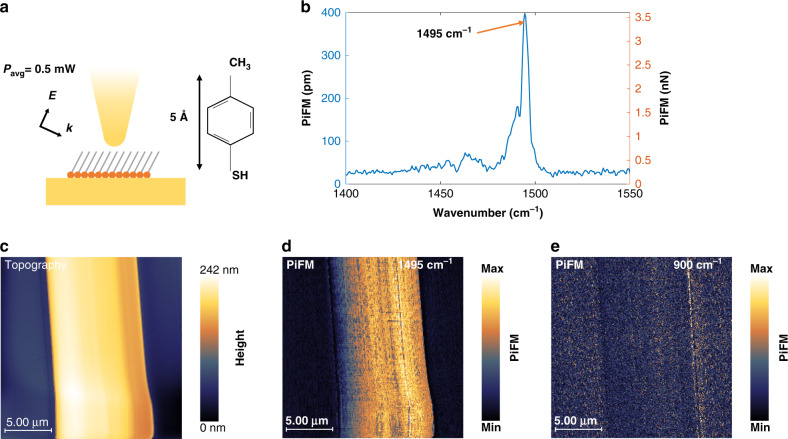
Monolayer response. **a** is 4-MBT adsorbed on template-stripped gold. Excitation average power is 0.5 mW focused to 20-μm-diameter spot. **b** is point spectrum of 4-MBT showing resonance at 1495 cm^−1^. **c**–**e** are topography of gold island, PiFM image at 1495 cm^−1^, and PiFM image off resonance, respectively.

To demonstrate PiFM sensitivity to optical forces generated by molecular vibrational resonances of monolayer samples, 4-MBT self-assembled monolayer solution was prepared and TSG sample was immersed and left overnight in solution. The TSG is expected to be completely covered by a 4-MBT monolayer. Gold islands are generated by sonicating the TSG in ethanol until gold starts lifting off. Figure 4c shows the topography of the sample. The thickness of the monolayer is <5 Å^29^. The sample was excited with p-polarized light using QCL. Measured average power was 0.5 mW. The diameter of the focal spot is 20 μm, with incident angle of 30° measured from sample surface. The average tip–sample distance was controlled at *f*_2_ with dithering amplitude of 6 nm peak to peak. Set point was adjusted such that average tip–sample distance is approximately 9 nm (refer to Fig. 6b). Thus, minimum average tip–sample distance will be around 6 nm. QCL repetition rate was tuned such that the lower sideband *f*_2_ − *f*_m_ coincided with *f*_1_. The PiFM signal is enhanced by the quality factor (*Q*_1_) at *f*_1_, which is about 435 (see Supplementary Fig. 4). In addition to the mechanical enhancement, the silicon cantilever/tip was coated with 60-nm-thick gold to locally enhance the electromagnetic field.

Figure 4b shows the absorption spectrum of 4-MBT, which is centered at 1495 cm^−1^, with full width at half maximum of about 4 cm^−1^. This sharp absorption band is a typical signature of benzene ring mode. Figure 4c, d, respectively, show simultaneously recorded topography and PiFM images; Fig. 4e is PiFM image when 4-MBT is excited off resonance; it shows that the signal observed in Fig. 4b is not due to any possible crosstalk between topography and PiFM. Under similar experimental conditions, thermal expansion of a monolayer has been calculated numerically to be <3 pm with a temperature increase of <6 degrees^30,31^. Also, we have shown in the previous section that minimum detectable thermal expansion is about 32 pm—limited by our system noise level. Because the maximum measured signal for 4-MBT corresponds to oscillation amplitude of 392 pm peak–peak, and since *S* = 1 pm pm^−1^, the mono-molecular layer must expand 392 pm to generate our signal—almost 100% of its initial thickness! Such an expansion would imply heating the molecular layer by several 100s of degrees. We conclude that the observed PiFM signal clearly could not originate from thermal expansion. The findings also support the fact that any $F_{{\mathrm{th}}}^{{\mathrm{vdW}}}$ effect on the PiFM signal is negligible. In what follows, experiments were performed to study the effect of vibrational resonances on the cantilever dynamics and to unravel the actual contrast mechanism in IR-PiFM.

### Opto-mechanical damping

The sidebands in PiFM can originate from either amplitude modulation (AM) or frequency modulation (FM) of the second resonance *f*_2_ of the cantilever. In one analysis, it was considered to originate from a frequency modulation of *f*_2_ resonance^32^; a change in tip–sample interaction force (force gradient) leads to change in the effective spring constant, which in turn shifts *f*_2_ at the chopping frequency *f*_m_. Amplitude modulation of *f*_2_ is another way to generate sidebands. The excited molecule interacting with the tip exerts a damping force in the tip leading to a change in the cantilever effective damping constant (*γ*_eff_), which in turn amplitude modulates *f*_2_ at *f*_m_.

We conducted a series of experiments to determine whether the PiFM signal (mixed signal) detected at *f*_1_ is due to AM or FM modulation of cantilever second eigenmode resonance. In our experiments, first the cantilever was mechanically excited (by the dithering PZT) at a frequency slightly higher than *f*_2_ (*f*_2R_) and then excited at a frequency slightly lower than *f*_2_ (*f*_2L_). Note that *f*_2_ is used as feedback signal (to control the average tip–sample distance). The sample was 60 nm poly(methyl methacrylate) (PMMA) on glass. The laser was tuned to a PMMA resonance and modulated at *f*_m_ = *f*_2_ − *f*_1_. For attractive conservative optical interactions, *A*_2_ is expected to decrease when cantilever is mechanically excited at *f*_2R_ and increase when excited at *f*_2L_; this is because the attractive forces shift the frequency to a lower value. Therefore, because the two behaviors are opposite to each other, the expected relative phase $\left( {\varphi _{{\mathrm{rel}}} = |\varphi _1^{f_{2{\mathrm{R}}}} - \varphi _1^{f_{2{\mathrm{L}}}}|} \right)$ measured for PiFM signal is expected to be about 180° out of phase. For dissipative optical interactions, the amplitude *A*_2_ decreases for *f*_2R_ and *f*_2L_, hence *φ*_rel_ is about 0°. Thus the value of *φ*_rel_ can be used to understand the nature of the interaction. When we measured *φ*_rel_ of the PiFM signal at *f*_1_ using a lock-in amplifier, we discovered that *φ*_rel_ was nearly 0° (see Supplementary Fig. 5) indicating that our PiFM signal contrast was originating from AM rather than FM modulation of the cantilever second eigenmode.

**Fig. 5:**
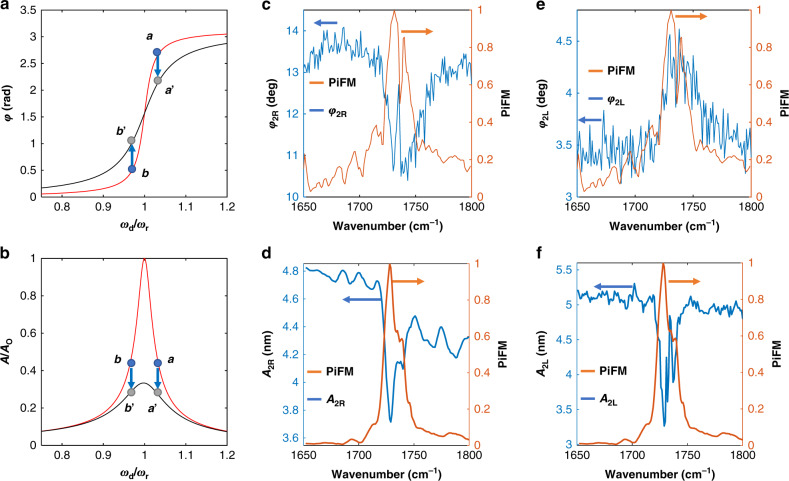
Opto-mechanical damping. **a**, **b** are phase and amplitude of high Q (red) and low Q (black) harmonic oscillator. Change in the phase (*φ*_2_) and amplitude (*A*_2_) of the second mechanical mode (blue line) across PMMA absorption band (orange line) centered at 1733 cm^−1^ for right excitation (**c**, **d**) and left excitation (**e**, **f**). Sample is 60-nm-thick PMMA on glass. The phase measurements were conducted using standard PiFM mixing mode, while amplitude measurements were conducted with the feedback loop open and the wavenumber scanned rapidly through resonance as explained in the text.

In order to confirm our finding by direct measurement, we perform the following experiment. The cantilever was mechanically excited at *f*_2R_. The tip was approached and engaged with the sample. The feedback loop was opened, and the laser wavelength was rapidly swept across PMMA absorption band centered at 1733 cm^−1^; the oscillation amplitude (*A*_2R_) at *f*_2R_ was recorded and compared with the point spectrum taken earlier for the same sample but with the control loop closed. The acquisition time needed to be fast enough to minimize thermal drift during data acquisition. In addition, laser modulation frequency *f*_m_ was set at 1 MHz so that it did not excite any cantilever eigenmodes (see Supplementary Fig. 6)—i.e., in these studies, we can consider the laser to be behaving essentially as a continuous wave source of energy. Our experiments revealed that the mechanical oscillation amplitude of the cantilever *A*_2R_ was damped as the laser was scanned through the PMMA resonance! (Fig. 5d). The experiment was repeated again with excitation frequency at *f*_2L_ with exactly the same result (Fig. 5f). Figure 5a. b show the expected phase and amplitude response of two harmonic oscillators with different quality factors. If photoinduced force was dissipative, the predicted amplitude behavior for *f*_2R_ and *f*_2L_ is shown as a transition from *a* to *a*’ and from *b* to *b*’. As mentioned, *A*_*2*_ decreases in both cases as shown in Fig. 5b, d, f, in contrast to what is expected from a conservative force. The change in *Q*_2_ is evident and it tracks the change in the point spectrum.

Another piece of evidence that demonstrates the dissipative nature of photoinduced force is shown in the phase measurements of Fig. 5c, e. The phase of *f*_2_ (*φ*_2_) was recorded while the optical wavenumber was rapidly swept through resonance. Here the experiment is done with the feedback loop closed. According to Fig. 5a, we should expect to observe a decrease in the phase for *f*_2R_ and an increase at *f*_2L_ as verified in Fig. 5c, e. The change in *φ*_2R_ is 3° at *f*_2R_ and the change in *φ*_2L_ is 1° at *f*_2L_. We note that the phase measurements and point spectrum were simultaneously recorded for the 60 nm PMMA film on glass.

As a further confirmation, we perform a direct measurement of the quality factor of the first mechanical mode (*Q*_1_). Here we have controlled the tip–sample gap using the second mechanical mode. The first mechanical mode is free (i.e., the amplitude and phase are not restricted by the feedback loop). Once we engaged with the sample, we excited the first eigenmode using dithering piezo to measure its quality factor with laser ON and OFF (i.e., both eigenmodes are excited mechanically using the dithering PZT). Similar to the previous experiment, we set *f*_m_ = 1 MHz. To measure *Q*_1_ at different average tip–sample distances, the frequency of the applied signal to the dithering PZT is tuned across *f*_1_ and the oscillation setpoint of the feedback loop is lowered. For each setpoint, we measure *Q*_1_ when we excite the sample on resonance (laser ON) and when the laser is OFF. *Q*_1_ decreases at a faster rate when sample is excited ON resonance versus when laser is OFF (see Supplementary Fig. 9).

Based on all our experiments, we conclude that a change in *γ*_eff_ of the cantilever rather than a change in its effective spring constant is the dominant contrast mechanism in PiFM. The intensity modulated excitation source modulates *γ*_eff_ at *f*_m_, which in turn generates the mixing signal measured at *f*_1_ = *f*_2_ − *f*_m_.

Consider the tip–sample ensemble as spring-dashpot-mass system; the oscillation of the cantilever normal to sample surface (**z** direction) can be approximately described by a non-linear, second-order differential equation

1 $$m\frac{{{\mathrm{d}}^2{\mathbf{z}}}}{{{\mathrm{dt}}^2}} + m\gamma _{{\mathrm{eff}}}\frac{{{\mathrm{d}}{\mathbf{z}}}}{{{\mathrm{dt}}}} + k{\mathbf{z}} = {\mathbf{F}}_{\mathbf{d}} + {\mathbf{F}}_{{\mathbf{int}}} + {\mathbf{F}}_{{\mathbf{opt}}},$$

where *m*, *k*, and *γ*_eff_ are the effective mass, stiffness, and damping constant, respectively. **F**_**int**_, **F**_**opt**_, and **F**_**d**_ are the nonlinear vdW tip–sample interactions, near-field tip–sample optical interactions, and deriving force, respectively. Velocity-dependent dissipative tip–sample interactions such as viscoelasticity and adhesion hysteresis are significant when imaging soft samples, such as polymers. Therefore, we propose that the optical energy absorbed by the sample could induce a measurable change in one or both of these dissipative channels leading to the observed opto-mechanical damping. Because viscoelasticity and adhesion hysteresis are velocity dependent^33^, we assume that the opto-mechanical damping is velocity dependent; thus the effective damping constant is the sum of the opto-mechanical damping constant *γ*_opt_ and the non-optical damping constant *γ*_cant_ and *γ*_ts_:

2 $$\gamma _{{\mathrm{eff}}} = \gamma _{{\mathrm{ts}}} + \gamma _{{\mathrm{cant}}} + \gamma _{{\mathrm{opt}}}.$$

In our experiments, *f*_2_ ~ 1.65 MHz and *Q*_2_ ~ 551. Then the damping coefficient $m_2\gamma _{{\mathrm{eff}}} = k_2\left( {\omega _0Q_2} \right)^{ - 1}$ (where *m*_2_ is the effective mass at *f*_2_) is approximately 65 nN s m^−1^. From Fig. 5e, f, we see that the cantilever oscillation is reduced by approximately 35% when the tip is stabilized 9 nm from the sample surface and the optical wavelength is tuned to the molecular resonance. We conclude that *γ*_opt_ is 0.35 × 65 or 22 nN s m^−1^ at molecular resonance. *γ*_opt_ reaches its maximum value when the molecule is driven at its optical resonance (i.e., at maximum optical polarization of the molecule).

### Theoretical modeling

In PiFM, we observe additional damping of the vibrating cantilever tip when the sample molecule is irradiated with photon energy corresponding to one of its molecular vibrational modes. We can model the increase due to opto-mechanical damping (Fig. 5) as follows^34^. The averaged mechanical power delivered to the cantilever by the dithering piezo at *ω*_d_ is given by

3 $$< P_{{\mathrm{in}}}^{{\mathrm{mech}}} > = \frac{1}{2}AA_{\mathrm{d}}k\omega _{\mathrm{d}}{\mathrm{sin}}(\phi ),$$

where *A* and *A*_d_ are oscillation amplitude of the cantilever and oscillation amplitude of the dithering piezo, respectively. *k* and *Φ* are stiffness of the cantilever and the phase difference between driving signal and cantilever oscillation, respectively. The power loss $< P_{{\mathrm{diss}}}^{{\mathrm{cant}}} >$ in the cantilever with quality factor *Q* and resonance frequency *ω*_d_ (primarily caused by air damping) is given by

4 $$< P_{{\mathrm{diss}}}^{{\mathrm{cant}}} > = \frac{1}{{2Q}}kA^2\omega _d.$$

If $< P_{{\mathrm{diss}}}^{{\mathrm{ts}}} >$ and $< P_{{\mathrm{loss}}}^{{\mathrm{opt}}} >$ are the averaged power loss due to non-optical tip–sample dissipative interactions and opto-mechanical damping of the cantilever, respectively, from power balance, we can write

5 $$< P_{{\mathrm{diss}}}^{{\mathrm{ts}}} > + < P_{{\mathrm{loss}}}^{{\mathrm{opt}}} > = < P_{{\mathrm{in}}}^{{\mathrm{mech}}} > - < P_{{\mathrm{diss}}}^{{\mathrm{cant}}} > .$$

Combining Eqs. (3)–(5) gives

6 $$< P_{{\mathrm{diss}}}^{{\mathrm{ts}}} > + < P_{{\mathrm{loss}}}^{{\mathrm{opt}}} > = \frac{{kA^2\omega _{\mathrm{d}}}}{{2Q}}\left( {\frac{{QA_{\mathrm{d}}{\mathrm{sin}}\left( \emptyset \right)}}{A} - 1} \right).$$

Solving for cantilever oscillation amplitude *A* gives

7 $$A = QA_{\mathrm{d}}{\mathrm{sin}}\left( \emptyset \right)\left( {\frac{{ < P_{{\mathrm{diss}}}^{{\mathrm{ts}}} > + < P_{{\mathrm{loss}}}^{{\mathrm{opt}}} > }}{{ < P_{{\mathrm{diss}}}^{{\mathrm{cant}}} > }} + 1} \right)^{ - 1}.$$

From Newton’s equation of motion for a damped oscillating cantilever, we can relate rate of dissipated mechanical power $< P_{{\mathrm{diss}}}^{{\mathrm{cant}}} >$ due to air damping to damping constant *γ*_cant_ through $< P_{{\mathrm{diss}}}^{{\mathrm{cant}}} >$ = 0.5 *mγ*_cant_*ω*_d_^2^*A*^2^ (where *m* is the effective mass of the cantilever); we can therefore write Eq. (5) in terms of *γ*_opt_ and *γ*_cant_.

8 $$A = QA_{\mathrm{d}}\sin (\emptyset )\left( {\frac{{\gamma _{{\mathrm{ts}}} + \gamma _{{\mathrm{opt}}}}}{{\gamma _{{\mathrm{cant}}}}} + 1} \right)^{ - 1}.$$

Equations (7) and (8) show that the cantilever oscillation amplitude decreases when the opto-mechanical damping constant *γ*_opt_ or the optically mediated tip–sample power dissipation increases—both will reach a maximum at optical resonance. Modulating the incident light intensity at *f*_m_ (*f*_m_ = *f*_2_ − *f*_1_) produces maximum sideband oscillation amplitude and therefore maximum signal at *f*_1_ when the sample is driven at one of its vibrational resonances. In addition, in order to get maximum sensitivity for detecting molecular resonance, we need to choose an AFM set-up with the lowest mechanical loss. S/N ratio will be greatly improved by working even in a rough vacuum where air damping would be significantly minimized.

The *z* component of the optical force *F*_tz_ acting on the tip with effective dipole moment *μ*_te_ can be written as^33^

9 $$F_{{\mathrm{tz}}} = \mu _{{\mathrm{te}}}\frac{{{\mathrm{d}}E_{{\mathrm{tz}}}}}{{{\mathrm{d}}z}}$$

or

10 $$F_{{\mathrm{tz}}}\,{\mathrm{d}}z = \mu _{{\mathrm{te}}}{\mathrm{d}}E_{{\mathrm{tz}}} = \alpha _{{\mathrm{te}}}\,E_{{\mathrm{tz}}}{\mathrm{d}}E_{{\mathrm{tz}}}.$$

Where *α*_te_ is the effective polarizability of the tip

11 $$F_{{\mathrm{tz}}}\frac{{{\mathrm{d}}z}}{{{\mathrm{d}}t}} = \alpha _{{\mathrm{te}}}E_{{\mathrm{tz}}}\frac{{{\mathrm{d}}E_{{\mathrm{tz}}}}}{{{\mathrm{d}}t}}.$$

The time-averaged mechanical power $< P_{{\mathrm{loss}}}^{{\mathrm{opt}}} >$ dissipated due to optical forces acting on the tip is

12 $$< P_{{\mathrm{loss}}}^{{\mathrm{opt}}} > = < F_{{\mathrm{tz}}}\frac{{{\mathrm{d}}z}}{{{\mathrm{d}}t}} > = 0.5{\mathrm{Re}}\left[ {\alpha _{{\mathrm{te}}}^ \ast E_{{\mathrm{tz}}}^ \ast {\mathrm{i}}\omega _0E_{{\mathrm{tz}}}} \right] = 0.5{\mathrm{Im}}\left[ {\alpha _{{\mathrm{te}}}^ \ast E_{{\mathrm{tz}}}^ \ast \omega _0E_{{\mathrm{tz}}}} \right],$$

where *ω*_0_ is the optical frequency and *E*_tz_ the *z*-component of the electric field at tip where

13 $$E_{{\mathrm{tz}}} = \left( {1 + \frac{{\alpha _{\mathrm{s}}}}{{2\pi (d + 2a_{\mathrm{t}})^3}}} \right)E_{\mathrm{i}}$$

and *α*_te_ is given by^34^

14 $$\alpha _{{\mathrm{te}}}\sim \alpha _{\mathrm{t}} + \frac{{\alpha _{\mathrm{t}}\alpha _{\mathrm{t}}\beta _{\mathrm{s}}}}{{16\pi (d + 2a_{\mathrm{t}})^3}}.$$

*α*_t_ and *α*_s_ are the polarizabilities of tip and sample, respectively, *d* is average tip–sample distance, *a*_t_ is tip radius, and *E*_i_ is the incident field.

To arrive at Eq. (14), we made the approximation $\left[ {\alpha _{\mathrm{t}}\left( {1 - \frac{{\alpha _{\mathrm{t}}\beta _{\mathrm{s}}}}{{16{\uppi}\left( {d + 2a_{\mathrm{t}}} \right)^3}}} \right)^{ - 1}} \right]\sim \left[ {\alpha _{\mathrm{t}} + \frac{{\alpha _{\mathrm{t}}\alpha _{\mathrm{t}}\beta _{\mathrm{s}}}}{{16\pi (d + 2a_{\mathrm{t}})^3}}} \right].$

Using Eqs. (10)–(14), $< P_{{\mathrm{loss}}}^{{\mathrm{opt}}} >$ can be written as

15 $$< P_{{\mathrm{loss}}}^{{\mathrm{opt}}} > \sim \frac{1}{2}{\Im} (a_{\mathrm{t}}^ \ast E_{\mathrm{i}}^2) + \frac{1}{2}{\Im} \left( {\frac{{\omega _{\mathrm{o}}a_{\mathrm{t}}^ \ast (a_{\mathrm{s}}^ \ast + a_{\mathrm{s}})E_{\mathrm{i}}^2}}{{2\pi (d + 2a_{\mathrm{t}})}}} \right) + \frac{1}{2}{\Im} \left( {\frac{{\omega _{\mathrm{o}}a_{\mathrm{t}}^ \ast a_{\mathrm{s}}^ \ast \beta _{\mathrm{s}}^ \ast E_{\mathrm{i}}^2}}{{16\pi (d + 2a_{\mathrm{t}})^3}}} \right),$$

where *β*_s_ = (*ϵ*_s_, −1)/(*ϵ*_s_, +1) is sample reflection coefficient, with *ϵ*_s_ the complex dielectric function of the tip. Tip polarizability *α*_t_ = *ξV*_t_(*ϵ*_t_, −1)/(*ϵ*_t_, +2), with *V*_t_ the effective tip volume, *ξ* is tip field enhancement factor when tip is far from surface, and *ϵ*_t_ is the complex dielectric function of tip. We have a similar expression for *α*_s_ with the parameters for sample replacing those of tip, except that *ξ* = 1 in the latter case. Based on our numerical simulations for the gold-coated tip, *ξ* ~ 10. The last term in Eq. (15) is the most dominant term.

**Fig. 6:**
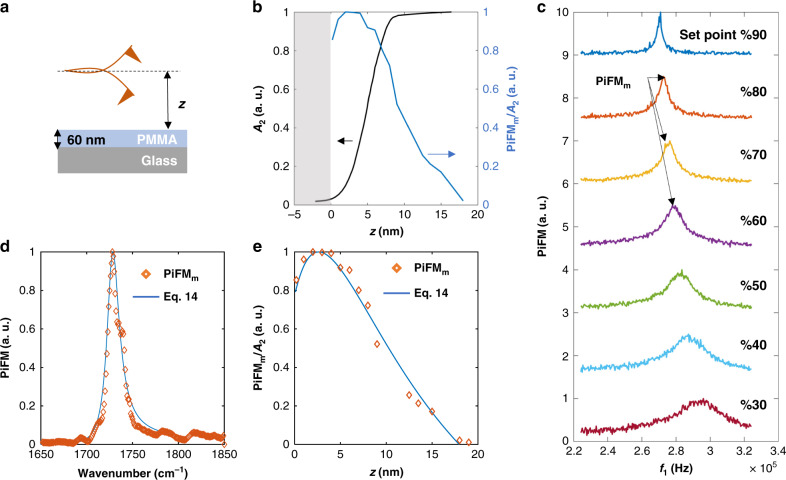
Experimental and theoretical results of opto-mechanical damping. **a** Schematic of the tip and sample. **b** Amplitude *A*_2_ measured at *f*_2_ (black line) and maximum value of PiFM signal (PiFM_m_) measured at *f*_1_ (blue line) as a function of average tip–sample distance. The shift in the peak value of *f*_1_ as a function of average tip–sample distance is tracked in **c** and normalized by the correspondent amplitude measured at *f*_2_. The set point for each curve in **c** is indicated as percentage of the amplitude of the cantilever oscillation at *f*_2_. **d**, **e** are comparisons of the experiment (diamond) and Eq. (15) and Eq. 16 (solid line) for point spectrum and approach curve, respectively.

Equation 15 was evaluated with the appropriate Lorentzian dielectric functions for PMMA and dielectric constants for the tip^35,36^. Figure 6d compares the experimental point spectrum (diamond symbol) with Eq. 15 (solid line) showing excellent agreement. For the PiFM approach curve, *f*_1_ was mechanically tuned to get the maximum PiFM signal for each setpoint (as shown in Fig. 6c). The tracked peaks were normalized to the corresponding *A*_2_. Figure 6b shows the PiFM signal and the corresponding *A*_2_ signal as a function of average tip–sample distance. We see that the PiFM signal is measurable up to 18 nm from sample surface. Figure 6e shows a fit to the experimental approach curve using Eq. (16). The data fits a *d*^−3^ dependence as expected up to a tip–sample distance of 5 nm. However, the PiFM signal shows a characteristic feature at molecular resonance; the signal increases monotonically as the tip approaches the sample but then decreases at the inflection point at 4 nm.

The inflexion point can be incorporated into our model by adding an additional complex distance −j*b* to the distance dependence^37,38^.

16 $$< P_{{\mathrm{loss}}}^{{\mathrm{opt}}} > \sim \frac{1}{2}\left[ {{\Im} (\omega _{\mathrm{o}}a_{\mathrm{t}}^ \ast )E_{\mathrm{i}}^2 + {\Im} \left( {\frac{{\omega _{\mathrm{o}}a_{\mathrm{t}}^ \ast (a_{\mathrm{s}}^ \ast + a_{\mathrm{s}})E_{\mathrm{i}}^2}}{{2\pi [(d + 2a_{\mathrm{t}} - {\mathrm{j}}b)]^3}}} \right) + {\Im} \left( {\frac{{\omega _{\mathrm{o}}a_{\mathrm{t}}^ \ast a_{\mathrm{t}}^ \ast \beta _{\mathrm{s}}^ \ast E_{\mathrm{i}}^2}}{{16\pi [(d + 2a_{\mathrm{t}} - {\mathrm{j}}b)]^3}}} \right)} \right].$$

While the overall force is attractive, this modification generates an additional distance-dependent repulsive term varying as −(*d* + 2*a*_t_)^−4^. Similar distance dependences have been predicted in models of the interaction energy of an atomic dipole oscillating close to a conducting sphere^39^. However, as we shall point out in the “Discussion” section, such a distance-dependent loss of signal at tip–sample spacings <9 nm can be due to different loss mechanisms.

The modified Eq. (16) was evaluated with tip radius *a*_t_ = 12 nm, sample radius *a*_s_ = 5 nm, and the fitting parameter representing loss strength *b* = 5.9 nm. The tip and sample radii were extracted experimentally by imaging grains on gold surface (see Supplementary Fig. 7). This modification generated an excellent fit between our model and the measured PiFM approach curve (Fig. 6e solid line).

## Discussion

Illuminating the cantilever with light could induce a temperature increase on the cantilever body. This increase in temperature can reduce the quality factor of the cantilever. A recent study showed that illuminating gold-coated cantilever near plasmonic resonance caused a measurable shift in the cantilever stiffness and a decrease in the quality factor^40^. Clearly this effect is not localized, and the temperature of the cantilever body has to increase for the quality factor to decrease. However, to extract nanoscale spectroscopic information of a sample, the opto-mechanical damping must originate and be localized within tip–sample volume of interaction, limited to the effective tip radius. The localization of the observed opto-mechanical damping is evident from Fig. 5d, f, where the change in *A*_2_ clearly follows the molecular resonance. Additionally, Fig. 6b shows that the opto-mechanical damping is measurable up to 20 nm from sample surface at best. This vertical confinement (normal to sample surface) is another piece of evidence that the observed opto-mechanical damping must originate from localized interactions.

The proposed mechanism of PiFM contrast is modeled as optically mediated damping of the viscoelastic or adhesion interactions between tip and sample. For example, when the tip approaches close to the sample, the high electric fields in the optical nanocavity will tend to align the molecules along the axis of the tip. As the tip is retracted from the sample, the molecules will tend to relax back toward their equilibrium position (position without any applied field). These periodic molecular relaxations can occur over a time comparable to the tip oscillation period but with a different phase shift as compared to the tip oscillation phase. In another scenario, the vibrating molecule could enhance adhesion hysteresis causing an additional mechanical loss. Note that both viscoelastic and adhesion loss mechanisms are velocity dependent^33^. Since opto-mechanical damping is assumed to originate from one or both mechanisms, modeling opto-mechanical damping as velocity-dependent dissipative process is justified.

Other possible mechanisms might contribute to the overall opto-mechanical damping. For example, Person et al. analyzed the effect of electron–hole (e–h) pair excitation on the life time of a vibrating point dipole near flat metal surface^38,41^. When a vibrating point dipole is located at a distance *d* from semi-infinite metal, inelastic interaction can occur, and part of the interaction energy is radiated away from the system through photon emission and the other part excites e–h pair in the metal, which is a nonradiative loss channel. This lossy interaction quenches the dipole moment of the vibrating molecule and is responsible for the repulsive term in the interaction Hamiltonian. The dipole strength was shown to increase then decrease as a function of *d*, showing a hump located a few angstroms from metal surface^37^, similar to what we observe in Fig. 6b. Note that modeling excited molecule with finite-dipole moment instead of point-dipole moment, and replacing flat metal surface with sharp metallic tips, spatially extends near-field gradient of the electromagnetic field, and including these two effects alters the distance dependence of the damping effect and could shift the hump a couple of nanometers away from sample surface.

Another mechanism for opto-mechanical loss could be due to the fact that molecule in the optical cavity is driven with very high fields. This will result in the energy in the driven molecular mode being distributed to other modes within the cavity resulting in reduced response at the driven molecular mode. There is some evidence of splitting of optical resonance spectra when driven with high optical power. This effect will require detailed investigation in a future article.

## Methods

### PiFM measurements

A commercial AFM (Molecular Vista Inc.) was used in non-contact mode for PiFM measurement. A pulsed QCL, from Block Engineering, was electrically triggered at *f*_m_ = *f*_2_ − *f*_1_ and focused to 20-μm-diameter spot; *f*_1_ and *f*_2_ are the first and second mechanical eigenmodes. QCL tuning range was from 800 to 1800 cm^−1^. The cantilever type used was PPP-NCHR-W 300 kHz from Nanosensors, where *f*_1_ = 264 kHz and *f*_2_ = 1.65 MHz. Quality factor (*Q*) of the first and second mechanical modes were *Q*_1_ = 435 and *Q*_2_ = 551, respectively.

### 4-MBT self-assembled monolayer

A TSG was prepared and immersed for 24 h in 0.3 mM 4-MBT (Sigma Aldrich) in ethanol. This was followed by sonicating the TSG till some of the gold surface started to lift-off; an island of Au were formed.

### PZT calibration

A heterodyne laser Interferometer was used to calibrate the vibrational expansion behavior of the PZT crystal (STEMINC). The arrangement consisted of a laser beam, beam splitters, Bragg cell, objective lenses, photodetector, high-gain low-noise amplifier, and a spectrum analyzer. HeNe laser source (632 nm) was used. The initial beam was split using a beam splitter. A portion was directed through a Bragg cell that shifted the optical frequency by 80 MHz. This beam acted as the reference beam of the interferometer. The other portion—the signal beam—was focused and reflected off the vibrating sample surface (the PZT crystal) generating a phase modulation proportional to the surface vibration amplitude.

## Supplementary information

Supplementary Information

## Data Availability

The data that support the findings of this study are available from the corresponding author upon reasonable request.
