# Supplementary material for: Observation of nanoscale opto-mechanical molecular damping as the origin of spectroscopic contrast in photo induced force microscopy
Source: Nat Commun. 2020 Nov 10;11:5691. doi: 10.1038/s41467-020-19067-3 (PMC7656459; doi:10.1038/s41467-020-19067-3)
Supplement: Supplementary file 1 — Supplementary Information [file 41467_2020_19067_MOESM1_ESM.pdf]

Observation of Nanoscale Opto-Mechanical Molecular Damping as the Origin of Spectroscopic Contrast in Photo Induced Force Microscopy

Almajhadi M. A. et al.

Supplementary Note 1: Photoacoustic signal as a function of air pressure.

When modulated light is incident on a sample surface, absorbed light heats up the sample causing a surface dilation and the gas in contact with it also heats up. The modulated pressure changes in the gas generate acoustic waves[1]. Microcantilevers have been used to detect the photoacoustic pressure waves[2]. In our experiments we show that in our system, photoacoustic pressure waves in the gas can be detected by the cantilever for the case of relatively thick samples. The sample used was a 500 nm Poly(methyl methacrylate) (PMMA) on glass substrate. The sample was excited at $f_m = f_1$ (direct PiFM mode). The, cantilever oscillation measured at the 1st eigenmode was used to measure the photoacoustic wave. We were able to detect a global photoacoustic effect originating from the focused infrared beam at the sample surface Supplementary Figure 1b, when the tip was retracted a few μm from sample. Photoacoustic signal should disappear when the system operates in vacuum. Thus, the tip-sample system is enclosed in a vacuum chamber and an air pump is used to decrease the pressure. Supplementary Figure 1d shows that the photoacoustic signal completely vanishes at 0.3 torr. The peak observed in Supplementary Figure 1b corresponds to the PMMA absorption band centered at 1733 cm^{-1}.

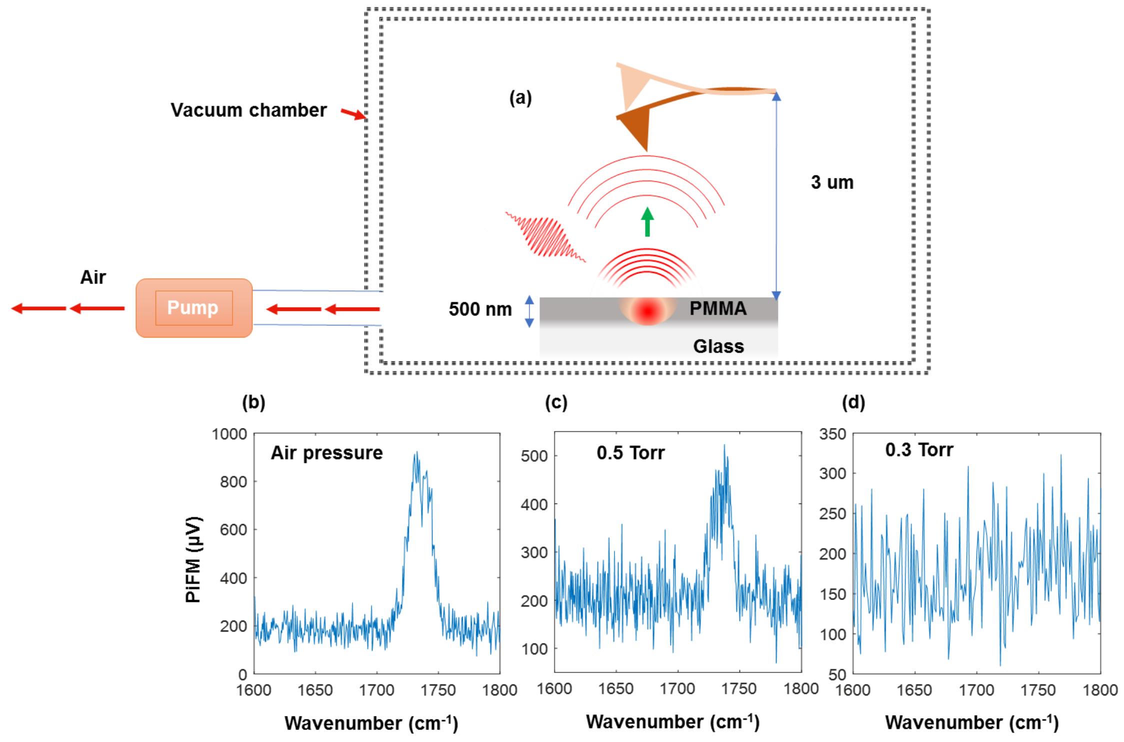

Supplementary Figure 1. Measurement of gas photo-acoustic waves while tip is retracted from sample by 3 μm. **a** 500 nm thick PMMA was excited by infrared laser source modulated at $f_m = f_1$ (direct PiFM mode), where tip-sample distance is about 3 μm. The tip-sample system were enclosed in a vacuum chamber and an air pump is used to decrease pressure. **b**-**d** Photoacoustic signals at 1.7 Torr, 0.5 Torr, and 0.3 Torr respectively

Supplementary Note 2: Interferometric setup for piezo calibration.

A heterodyne laser Interferometer was used to calibrate the vibrational expansion behavior of the PZT crystal. The arrangement consisted of a laser beam, beam splitters, Bragg cell, Objective lenses, photodetector, high gain low noise amplifier (LNA) and a

spectrum analyzer. The experimental set up is shown in Supplementary Figure 2. We use a HeNe laser source for our experiment. The initial beam was split using a beam splitter. A portion was directed through a Bragg cell that shifts the optical frequency by 80 MHz. This beam acted as the reference beam of the interferometer. The other portion – the signal beam – was focused and reflected off the vibrating sample surface (the PZT crystal) generating a phase modulation proportional to the surface vibration amplitude. The signal beam and reference beam were interfered at the photodiode to generate a carrier frequency at 80 MHz and sidebands corresponding to the piezo vibration frequency in the detected photocurrent. The electronics consisted of a photodetector, very high gain LNA and a spectrum analyzer. The spectrum analyzer, showed a large peak at the beat frequency centered at f_{c} = 80 MHz and smaller peaks at $f_{\mathrm{c}} \pm m f_{\mathrm{m}}$, where f_{m} is the frequency of piezo vibration and m is an integer number ranging from 0 to infinity. The ratio of the heights of the various sidebands can be used to precisely determine the amplitude of the vibration of the PZT crystal. If the vibration amplitude is very small compared with the laser wavelength (0.6328 μm), then the dominant sideband is the first one (i.e. m = 1). In that case, the relation between the amplitude of the first sideband A' and the carrier frequency A is given by a rather simple expression

$$\frac{A\prime}{A} = k\delta_{\mathrm{s}} \qquad (1)$$

Where k = 2π/λ , λ being the He-Ne wavelength and, δ_{s} the amplitude of PZT vibration. The PZT vibration amplitude varies linearly with the applied voltage. In our experiment, we increase the ac voltage stepwise and calculated the resulting expansion using Eq. 1. The calibrated periodic vibration amplitude of the PZT crystal at different frequencies

and at different applied voltages was used to simulate the thermal expansion induced by photo-absorption in the PiFM

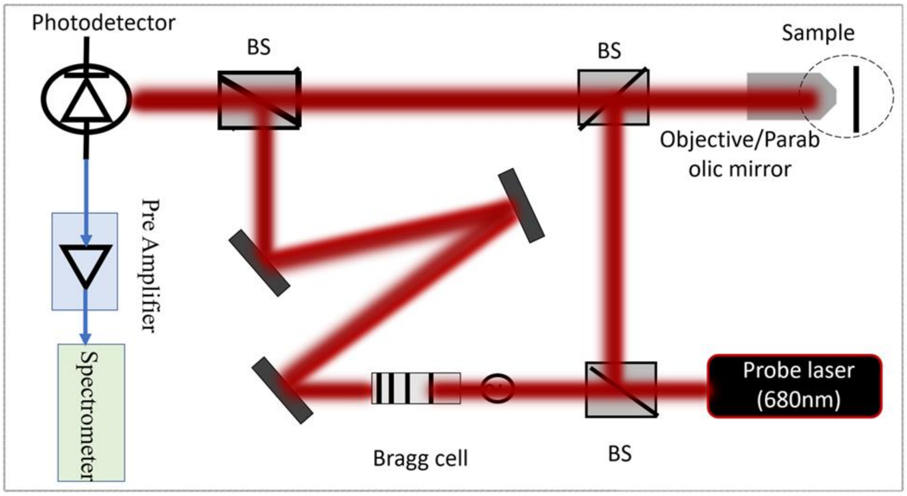

Supplementary Figure 2. Interferometric setup for calibrating the PZT Crystal.

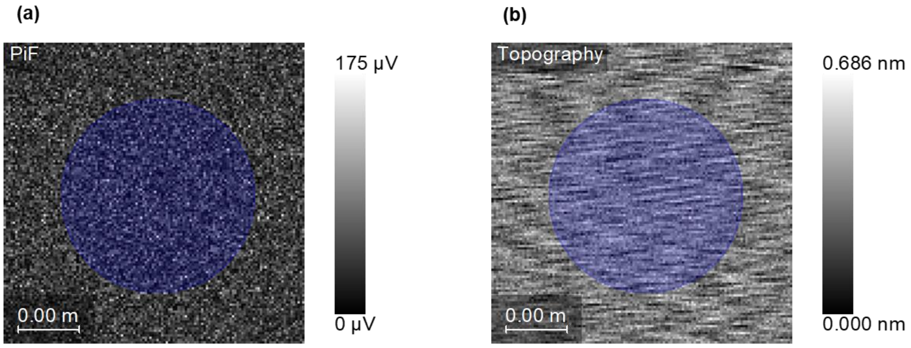

Avg (uV)	43.3
Min (uV)	0.476
Max (uV)	174.4

Avg (nm)	0.3313
Min (nm)	-0.0111
Max (nm)	0.6996

Supplementary Figure 3. Noise level measurement. Measured noise at 1st **a** and 2nd **b** mechanical modes. The image is for one point (sample is not moving in x-y direction).

The averaged noise is taken from the shaded area in the images. Note that the noise level at the 2nd mechanical mode is about 300 pm.

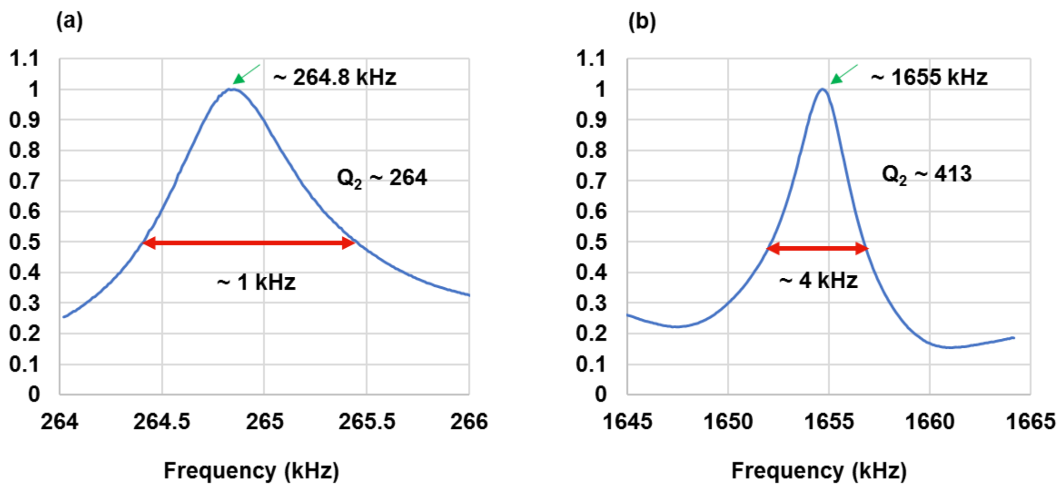

Supplementary Figure 4. Quality factor measurement. Measured frequency and quality factors of the 1st **a** and 2nd **b** mechanical modes of the cantilever. The cantilever was excited using dithering piezo. The 1st and 2nd eigenmodes are extracted by tuning the excitation frequency of the dithering piezo.

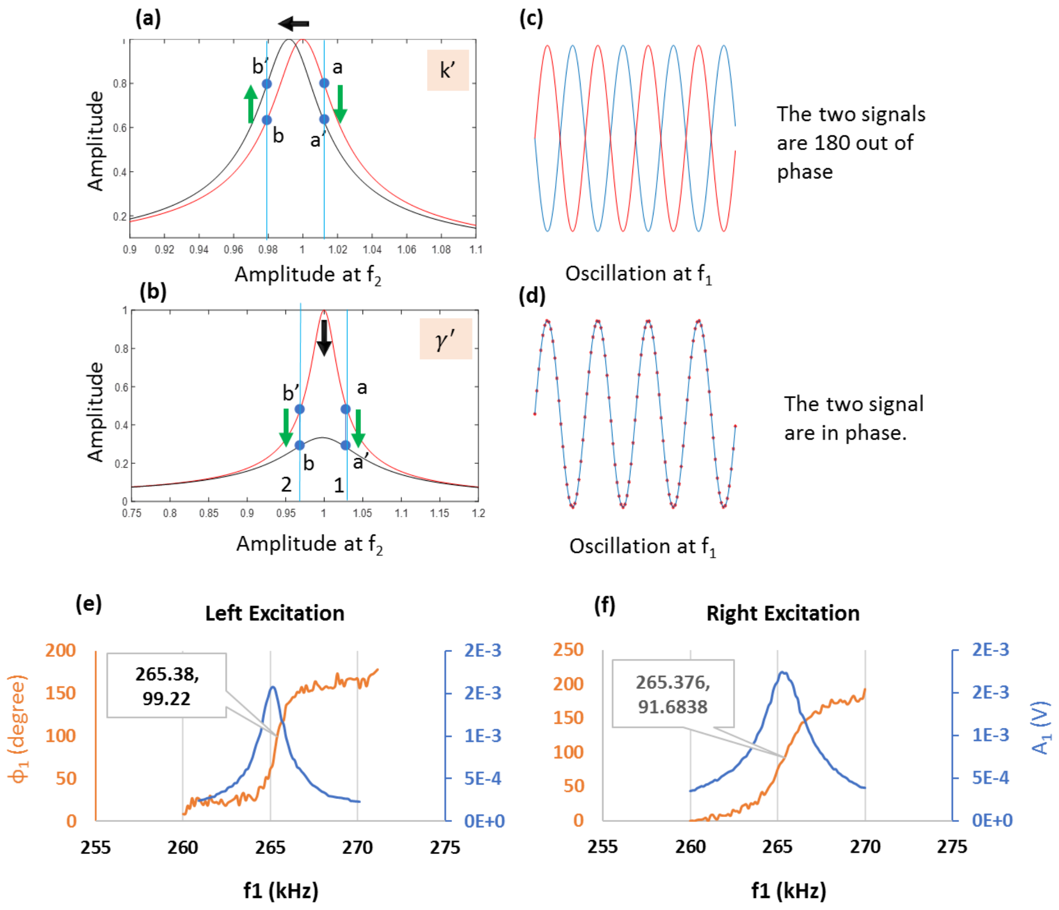

Supplementary Figure 5. Phase measurement comparison between left and right excitation. Amplitude change of the 2nd mechanical mode due to conservative **a** and dissipative **b** interactions. The expected relative phase between f_{2R} (a-a’) and f_{2L} (b-b’) excitations, measured at the 1st mechanical mode is shown in **c** and **d**. Phase measurement of the 1st mechanical mode for left excitation **e** and right excitation **f.** The phase and amplitude are measured using lock-in amplifier.

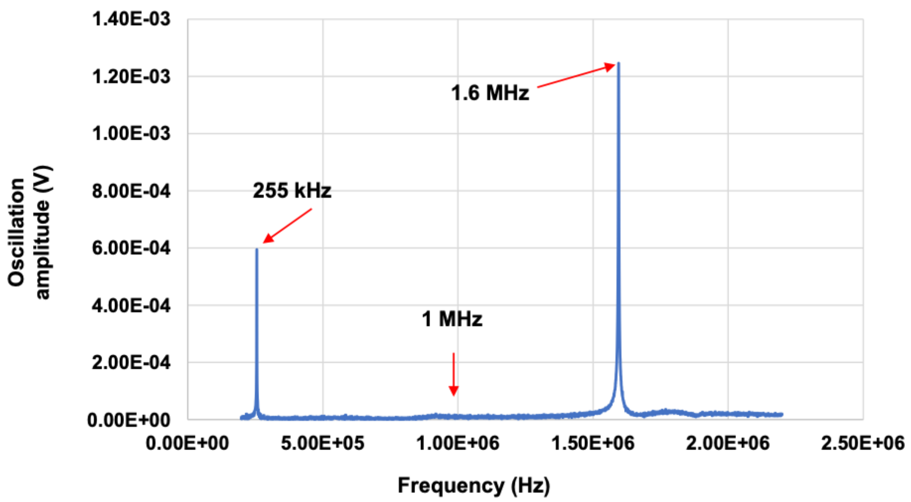

Supplementary Figure 6. 1st and 2nd mechanical modes of the cantilever used in this paper. Note that at 1 MHz there is no cantilever oscillation.

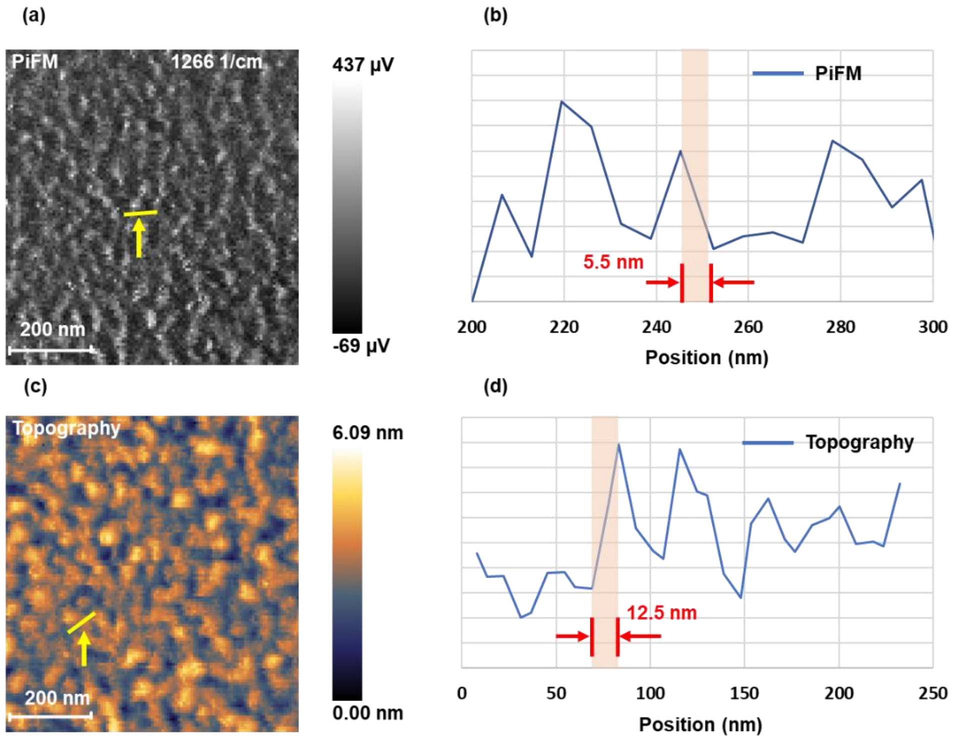

Supplementary Figure 7. Tip and sample effective radius measurement. Imaging grain size of deposited gold on Si substrate using e-beam evaporation. **a** and **c** are PiFM

image (excitation wavenumber is 1266 cm^{-1}) and the topography of the Au grains. **b** and **d** are line trace (yellow line) across the smallest feature, which shows special resolution of about 5.5 nm for PiFM and 12.5 nm for topography. PiFM signal is used to estimate sample radius (a_s) and topography channel is used to estimate tip radius (a_t).

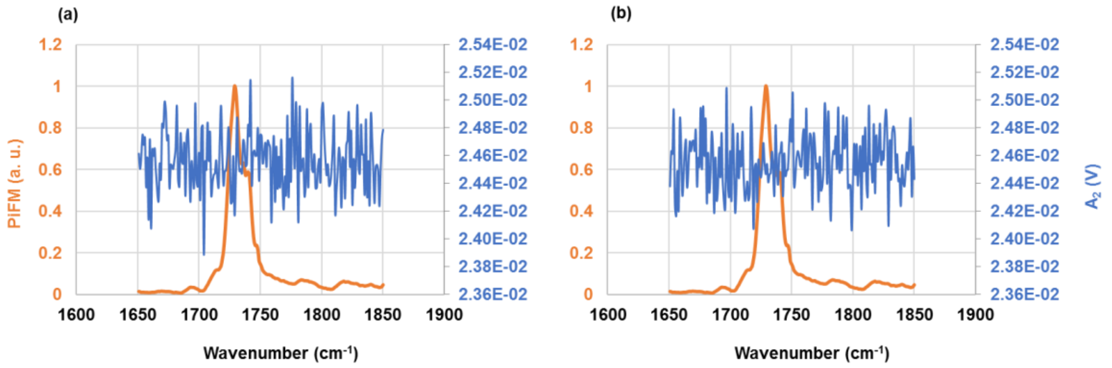

Supplementary Figure 8. Change in the cantilever oscillation amplitude A_2 (blue line) across PMMA absorption band at 1733 cm-1 (orange line). **a** Amplitude change for right excitation and **b** for left excitation. Chopping frequency f_m = 1MHz.

Supplementary Note 3: Change in A_1 as a function of averaged tip-sample distance

Supplementary Figure 9 (see below) demonstrates the effect of opto-mechanical damping (OMD) as a function of tip-sample averaged distance. For each averaged tip-sample distance, we will measure the quality factor of the 1st eigenmode of the cantilever with light and without light. Note that ‘with light’ means exciting the sample on resonance ‘without light’ means turning the laser OFF. Here, we have controlled the tip-sample gap using the 2nd eigenmode. The 1st mechanical mode is free. Once we engaged with the sample, we excite the 1st eigenmode using dithering piezo to measure its quality factor with laser on and off (i.e. both eigenmodes are excited mechanically

using the dithering PZT). To avoid modulation induced by the laser, we tuned the modulation frequency of the laser to 1 MHz (no cantilever oscillation at 1 MHz see Supplementary Figure 6). To measure the quality factor of f_1 (Q_1) for different tip-sample averaged distances, the frequency of the applied signal to the dithering PZT is tuned across f_1. The measured quality factor is shown in Supplementary Figure 9 solid circles. For each tip-sample distance, we measure Q_1 when we excite the sample on resonance (laser on) and laser off. The sample is 60 nm PMMA on gold; Excitation wavenumber is 1733 cm^{-1}. By comparing the quality factor, Supplementary Figure 9 clearly shows the effect of opto-mechanical damping.

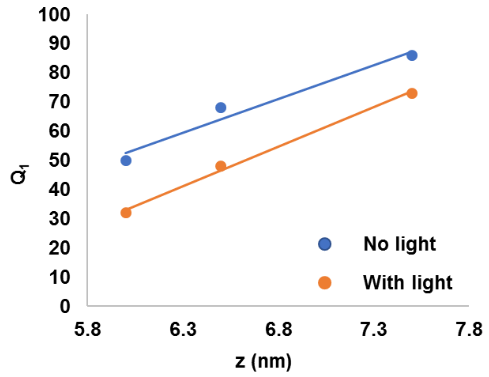

Supplementary Figure 9. While tip is engaged with the sample, change in the quality factor of f_1 (Q_1) as a function of tip-sample distance is measured. The Q is measured for each tip-sample distance as shown (solid circles) with light (orange) and without light (blue).

Supplementary Table 1. Definition of the most important symbols used in this paper

ω_d	Excitation frequency that goes to the dithering piezo
A_d	Oscillation amplitude of the dithering piezo
A	Oscillation amplitude of the cantilever
φ	Phase angle between the driving force and cantilever oscillation

k	Cantilever stiffness
$< P_{\mathrm{in}}^{\mathrm{mech}} >$	Average power delivered mechanically
$< P_{\mathrm{diss}}^{\mathrm{cant}} >$	Frictional loss due to tip-sample dissipative interaction and fluid surrounding the cantilever.
$< P_{\mathrm{loss}}^{\mathrm{opt}} >$	Frictional loss due to near-field optical interaction
γ_{cant}	Damping constant relates to the rate of the dissipated mechanical power due to cantilever interacting with the sample and the environment
γ_{opt}	Opto-mechanical damping constant relates to the rate of the dissipated mechanical power due to optical near-field interaction
F_{tz}	The z component of the optical force acting on the tip
$\boldsymbol{\mu}_{\mathbf{te}}$	Effective dipole moment of the tip
α_{te}	Effective polarizability of the tip
$\mathbf{E}_{\mathbf{i}}$	Incident electric field
E_{tz}	z-component of the local electric field at tip
ω_0	Optical frequency
α_t	Tip polarizability
α_s	Sample polarizability
a_t	Tip radius
a_s	Sample radius
d	The average tip-sample distance

Supplementary References:

1. Tam, A. C. Applications of photoacoustic sensing techniques. *Rev. Mod. Phys.* **58**, 381–431 (1986).
2. Adamson, B. D., Sader, J. E. & Bieske, E. J. Photoacoustic detection of gases using microcantilevers. *J. Appl. Phys.* **106**, 114510 (2009).
